# Adiponectin improves NF-κB-mediated inflammation and abates atherosclerosis progression in apolipoprotein E-deficient mice

**DOI:** 10.1186/s12944-016-0202-y

**Published:** 2016-02-18

**Authors:** Xuemei Wang, Qingjie Chen, Hongwei Pu, Qin Wei, Mingjun Duan, Chun Zhang, Tao Jiang, Xi Shou, Jianlong Zhang, Yining Yang

**Affiliations:** Xinjiang Key Laboratory of Medical animal Model Research, Clinical Medical Research Institute of First Affiliated Hospital of Xinjiang Medical University, No.137 South Liyushan Road, Urumqi, 830011 Xinjiang China; Department of Science and Research Education Center, First Affiliated Hospital of Xinjiang Medical University, No.137 South Liyushan Road, Urumqi, 830011 Xinjiang China

**Keywords:** Adiponectin, Atherosclerosis, Inflammation, NF-κB signaling pathways

## Abstract

**Background:**

Atherosclerosis is a common pathological basis of cardiovascular disease. Adiponectin (APN) has been shown to have an anti-atherosclerosis effect, and the underlying mechanisms, however, are largely unknown. Nuclear factor κB (NF-κB) has also been regarded as a proatherogenic factor, mainly because of its regulation of a variety of the proinflammatory genes linked to atherosclerosis. It was hypothesized that the inhibitory effects of adiponectin on the atherosclerosis is through the inhibition of NF-κB signaling pathway.

**Methods:**

We injected adenovirus of Ad-eGFP virus (control group) or the same amount of Ad-APN-eGFP virus (APN group) in ApoE^-/-^ mice tail-intravenously. Blood samples and aorta were executed at 0 day, 4, and 8 week of high-fat diet feeding. Histopathological changes of aortic arch root were detected. Levels of TC, TG, HDL-C, LDL-C were measured. Adiponectin and Matrix metalloproteinases-9 (MMP-9) concentration were detected by enzyme-linked immunosorbent assay. Gene and protein levels of adiponectin, eNOS, IL-6, MCP-1,VCAM-1, and other inflammatory factors were determined. Adiponectin, NF-κB p65 in aortic arch root were determined by immunofluorescence and western blot.

**Results:**

Transduction of Ad-APN inhibited the formation of atherosclerotic plaque in aorta when compared with control group. The lesion formation in aortic arch root was inhibited significantly (*P* < 0.01). Lesion lumen ratio decreased significantly (*P* < 0.001). The expression of adiponectin attenuated the increases of serum TC (*P* < 0.001), TG (*P* < 0.001), and LDL-C (*P* < 0.001) induced by the high-fat diet, and the increase in body weight (*P* < 0.05). As increasing serum adiponectin, the levels of MMP-9 were significantly decreased (*P* < 0.05). The exogenous adiponectin increased the gene expression of the anti-inflammatory factors eNOS (*P* < 0.05) and IL-10 (*P* < 0.001), and reduced the gene expression of inflammatory factors tumor necrosis factor-α (TNF-α) (*P* < 0.001), IL-6 (*P* < 0.001), VCAM-1 (*P* < 0.05), respectively. Adiponectin effectively inhibited the activation of NF-κB pathway and the expression of NF-κB nuclear protein p65.

**Conclusions:**

Adiponectin may protect the aorta from atherosclerotic injury by reducing inflammation. The molecular mechanism may involve inhibited the expression of downstream components of NF-κB and its transcription factors.

## Background

Cardiovascular disease is one of the leading causes of death worldwide [1]. Atherosclerosis is one important cause of cardiovascular disease. Atherosclerosis is characterised by the accumulation of lipid-laden macrophages in atherosclerotic lesions and occurs preferentially at arterial branching points, which are prone to inflammation during hyperlipidaemic stress [2]. Inflammation mediates its effects on atherosclerosis both through modulation of traditional risk factors and by directly affecting the vessel wall. Treatments such as TNF-α inhibitors might have a beneficial effect on cardiovascular risk [3]. Further knowledge of the predictors of cardiovascular risk, the effects of early control of inflammation and of drug-specific effects are likely to improve the recognition and management of cardiovascular risk in patients with atherosclerosis.

Adiponectin, also referred to as ACRP30 (adipocyte complement-related 30 kDa protein), is an specific adipokine secreted by adipose tissue [4]. Adiponectin exerts its biological effects via binding to two structurally and functionally distinct, G protein-coupled, seven-transmembrane receptors, adiponectin receptors 1 and 2 (AdipoR1 and AdipoR2) [5]. Chronic inflammatory states in obesity, obesity-linked insulin resistance, and diabetes are associated with reduced expression of both adiponectin and its cell surface receptors [6]. Clinical studies indicate a close association between low plasma adiponectin levels and atherosclerosis [7]. In addition, plasma levels of C-reactive protein (CRP) and IL-6 levels are both negatively correlated with plasma adiponectin levels [8]. In vitro experiments showed that adiponectin reduces the expression of class A scavenger receptor(SR-A) in human monocyte-derived macrophages and prevents macrophage foam cell formation. Adiponectin inhibits endothelial synthesis of IL-8 in human aortic endothelia cells stimulated with TNF-α [9]. Of importance, ApoE^-/-^/APN^-/-^ double-KO mice show accelerated atherogenesis, accompanied by increased T lymphocyte accumulation in atherosclerotic lesions, compared to ApoE^-/-^ mice [10]. Also there were experiments showed that the actions of adiponectin on the cardiovascular system are complex and multifaceted, with a minimal direct impact on atherosclerotic plaque formation in preclinical rodent models [11]. In this study, atherosclerosis mice model was established, intervented with the adenovirus expressing mice adiponectin DNA(Ad-APN), to investigate the effect of adiponectin prevention in atherosclerosis. And investigate the effect of adiponectin on the protein p65 in NF-κB signaling pathway and the downstream of the inflammatory cytokines expression. Laying the theoretical basis for treatment of atherosclerosis with adiponectin.

## Methods

### Mouse model of atherosclerosis

Male ApoE^-/-^ mice aged 12 weeks were used in this study as animal model of atherosclerosis. All procedures related to the animal experiments were approved (IACUC-20130709010) by the Animal Ethics Committee of First Affiliated Hospital of Xinjiang Medical University. High-fat diet (Jiangsu Medicine Co., Ltd. Nanjing, China) containing 35 % fat, 45 % carbohydrates, and 1.25 % cholesterol. Adenovirus vector containing the gene for full length mouse adiponectin was ordered from Shenzhen Byrne Biotechnology Co., Ltd. 120 male ApoE^-/-^ mice aged 12-week were randomly and evenly assigned into two groups (60 mice per group), and were fed with a high-fat diet to induce atherosclerosis. At 0 day, 2, 4, and 6 week of high-fat diet feeding the 2 groups of mice were injected tail-intravenously with either 100 μl (3.0 × 10^8^ p.f.u) of Ad-eGFP virus (control group) or the same amount of Ad-APN-eGFP virus (APN group). Blood samples and aortas tissues were harvested at 0 day, 4, and 8 week.

### Pathological detection of vascular tissue

Aortas were carefully excised from mice and examined for immunohistology and characterization of atherosclerotic lesions. Each was then fixed with paraformaldehyde and subjected to common dehydration to render the sample transparent. After that, paraffin embedding was performed and the samples were sliced. For the aortic root, Oil red O staining was used to detect the surface lesion percentage; Hematoxylin-eosin (HE) staining was used to detect the pathological alterations; Masson staining was used to evaluate the collagen content and fibrous cap thickness of the plaque area; Immunohistochemical approach was used to detect the overexpression of adiponectin, Immunohistochemical approach was used to detect the proportion of macrophages and smooth muscle cells; Analysis was performed on the Image J to determine the area of damage caused by atherosclerosis in the aorta and the lesion rate of arterial lumen [12].

### Measurement of blood parameters

A whole blood sample was held for 30 min at room temperature to allow clotting. The sample was centrifuged at 3,000 *g* for 10 min at 4 °C. The serum was transferred in separate tubes without disturbing blood clots and stored at −80 °C [13]. Full automatic biochemical analyzer (FABA) (HITACHI 7020) was used to assess total cholesterol (TC), triglyceride (TG), high-density lipoprotein cholesterol (HDL-C), and low-density lipoprotein cholesterol (LDL-C). The double-antibody sandwich enzyme-linked immunosorbent assay (ELISA) method was used to determine the concentration of adiponectin and MMP-9 in the serum. An enzyme-labeled instrument (Bio-Rad Benchmark Plus), blood parameter measurement reagent (Roche Diagnostics (Shanghai) Ltd. Shanghai, China), adiponectin ELISA kit (America Biomerica Company) and MMP-9 ELISA kit (USA & Canada | R&D Systems, Inc) were used in the experiments.

### Determination of mRNA levels

Real-time fluorescence quantitative polymerase chain reaction(qPCR) test was used to assess gene expression. Total RNAs were extracted from the aorta of mice using Trizol (Invitrogen, California, USA) according to the manufacturer’s instructions. Then 1 μg DNase I-treated (Thermo, USA) total RNA was reverse-transcribed using Primer Script Strand cDNA Synthesis Kit (Promega, USA). Reverse transcription and real-time fluorescent quantization were performed according to the instructions provided. PCR device (BIO-RAD My Cycler), electrophoresis apparatus trophoresis (Liuyi Brand DYY-6D) and high speed freezing centrifugal (HC-3018R) were used. Samples were run in triplicate. The housekeeping gene β-actin was used for internal normalization. The mean SQ values of the target gene primers were compared to those of β-actin specific primers using the double standard curve method. Data are presented as fold changes of transcripts for the target gene normalized to β-actin compared with control mice. Primers were designed to amplify mouse adiponectin, eNOS, TNF-α, IL-6, VCAM-1, β-actin, IL-10. All primers were obtained from Takara (Table 1). The reaction was under the following reaction conditions: 35 cycles of 94 °C for 30 s, 55 °C for 30 s, and 72 °C for 30 s. A DNA purification kit was used (Tiangen Biotech Co., Ltd., China), cDNA was amplified using a QuantiFast SYBR® Green Real-time PCR Master Mix (Qiagen, GER) in 96-well optical reaction plates on an fluorescent quantization PCR device (CFX96) according to the manufacturer’s protocol. The cycling parameters were as follows: 95 °C for 5 min and then 40 cycles of 95 °C for 10 s, 60 °C for 30 s, followed by a melting curve analysis.

**Table 1:** Sequences of primers for quantitative real-time RT-PCR

Genes	Forward	Reverse
*β-actin*	5′- GCTCTTTTCCAGCCTTCCTT -3′	5′-CTTCTGCATCCTGTCAGCAA-3′
*APN*	5′- AGGTTGGATGGCAGGC -3′	5′-TCTCACCCTTAGGACCAAGAA-3′
*eNOS*	5′- TGTCTGCGGCGATGTCACT -3′	5′- CATGCCGCCCTCTGTTG -3′
*TNF-α*	5′-GTCCCCAAAGGGATGAGAAG-3′	5′- CACTTGGTGGTTTGCTACGA-3′
*IL-6*	5′-CCGGAGAGGAGACTTCACAG-3′	5′-TCCACGATTTCCCAGAGAAC-3′
*VCAM-1*	5′-GCCCTCACTTGCAGCACTAC-3′	5′-TCCTCACCTTCGCGTTTAGT-3′
*IL-10*	5′-GCTCTTACTGACTGGCATGAG-3′	5′-CGCAGCTCTAGGAGCATGTG-3′

### Immunofluorescence staining

Immunofluorescence staining was used to identify NF-κB p65 and adiponectin protein localization in sections of aortic root. Paraffin-embedded aortic, sections were incubated with 3 % H_2_O_2_, then for antigen repair and closure [13]. Sections were then incubated overnight at 4 °C with a NF-κB p65 antibody (America Thermo Fisher, 1:50, rabbit polyclonal), The sections were then incubated with goat anti-rabbit lgG, dylight™ 594 (America Thermo Fisher, 1:200, goat anti-rabbit IgG). Sections were counter-stained with DAPI to identify nuclei before mounting. Then counter-stained with confocal microscopy. The sections were used for sequential double immunofluorescence staining. Primary antibody for adiponectin (America Thermo Fisher, 1:250, mouse momoclonal) and secondary antibody dylight™ 488 (America Thermo Fisher, 1;200, goat anti-mouse IgG), von Willebrand factor (vWF; endothelial cell marker, Abcam, Cambridge, MA, 1:800, rabbit polyclonal) and the sections were then incubated with goat anti-rabbit lgG, dylight™ 594 (America Thermo Fisher, 1:200, goat anti-rabbit IgG). Sections were counter-stained with DAPI to identify nuclei before mounting. Then counter-stained with confocal microscopy.

### Measurement of protein expression by western blot analysis

For western blot (WB) analyses, whole aortas were separately in 200 μl lysis buffer (Tiangen Biotech Co., Ltd., China) at 1:100 ratios, adiponectin for total protein, and NF-κB p65 nuclear fractions were prepared using the NE-PER Nuclear Protein Extraction kit (Tiangen Biotech Co., Ltd., China) according to manufacturer’s instruction. Protein concentrations were assessed with the BCA protein assay kit (Thermo Scientific, Rockford, IL). Equal amounts of protein (5 μg) were separated by 7.5 % SDS-PAGE and transferred to nitrocellulose membranes (Bio-Rad). Membranes were blocked in 5 % nonfat milk in PBS with 0.1 % Tween20 or chemiluminescent blocker (Millipore) at room temperature for 1 h. Expression of adiponectin, NF-κB p65, and GAPDH protein was detected by WB analysis with the following primary antibodies at the given dilutions: adiponectin (America Thermo Fisher, 1:1000), NF-κB p65 antibody (America Thermo Fisher, 1:100), and GAPDH (Abcam, 1:3000). They were shaken with primary antibody for 2 h. They were incubated at 4 °C overnight. After hatching with 1:1000 diluted secondary antibody at 37 °C for 1 h, they were warm bathed with chemiluminescence reagent ECL and exposure, developing, and fixing were performed 1 min later. Quantity One software was used for analysis; the integral optical density value was calculated as the target protein divided by the internal reference GAPDH protein. The relative values of protein bands in each group were expressed as mean ± standard deviation (SD).

### Statistical analysis

Statistical analysis was performed with SPSS 22.0 software. After the numeric variable data were tested for normality and homogeneity of variance, all parameters determined in this study are presented as mean ± SD. The differences among groups were compared using General Linear Model-Univariate or One-way ANOVA. *P* < 0.05 was considered statistically significant difference.

## Results

### Overexpression of adiponectin inhibits atherosclerosis in ApoE^-/-^ mice

**Fig. 1:**
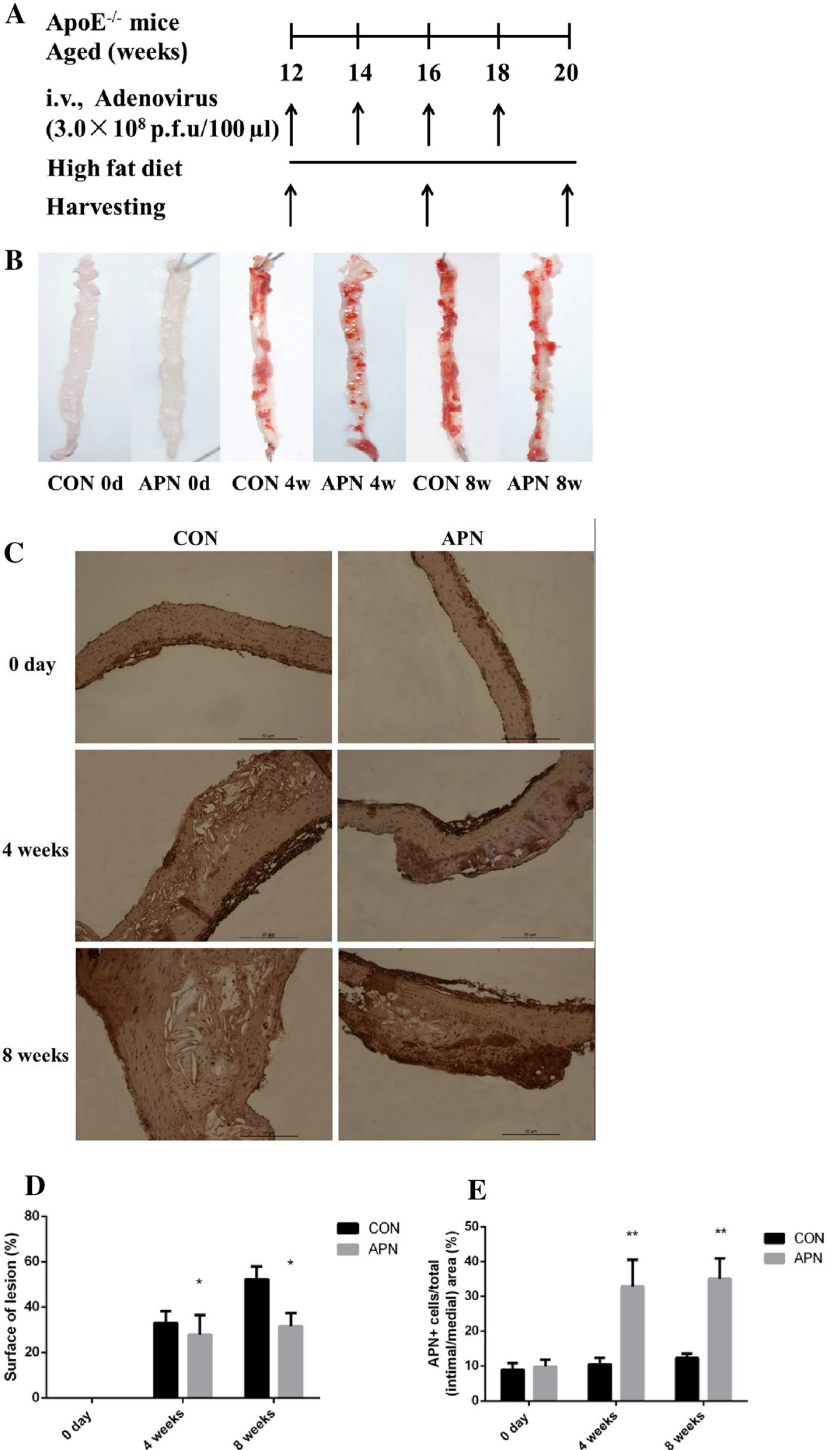
Overexpression of adiponectin inhibits atherosclerosis in ApoE^-/-^ mice **a** Schema of experimental procedure **b** Lesion areas shown were quantified using Oil-red O staining of the thoracoabdominal aorta **c** The overexpression of adiponectin of the aortic arch root by immunohistochemical approach (×200) **d** Surface of lesion in thoracoabdominal aorta (%) **e** the number of APN+ cells/% of total (intimal/medial) area. Notes: compared with CON group, *P < 0.01, **P < 0.001, *n* = 6, mean ± SD

**Fig. 2:**
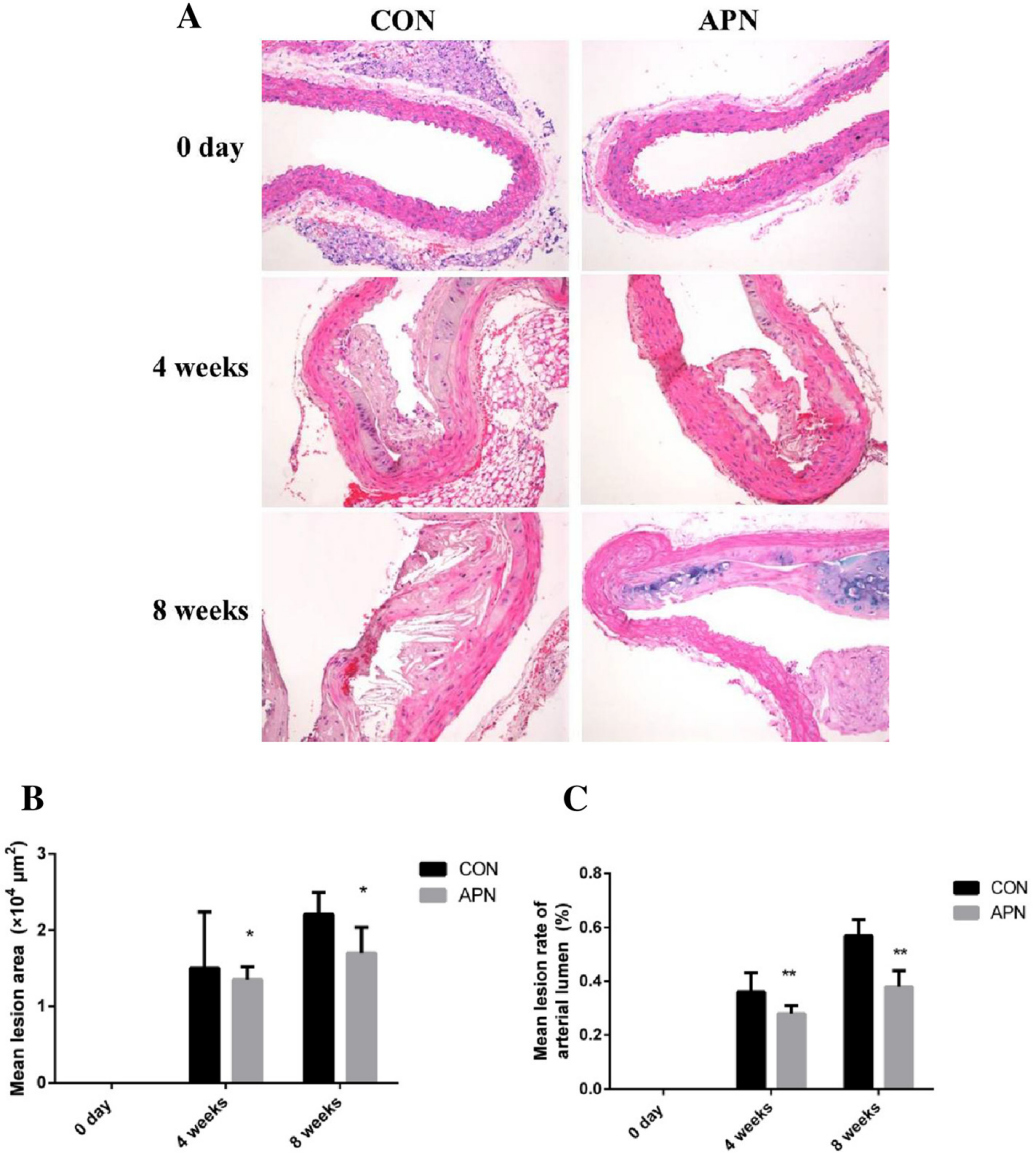
Mean lesion area and mean lesion rate of the aortic arch root **a** HE staining × 200 **b** The average lesion area of each mouse was determined from aortic root **c** The mean lesion rate. Notes: compared with CON group, *P < 0.01; **P < 0.001, *n* = 6, mean ± SD

The adenovirus mediated overexpression of adiponectin inhibited the formation of atherosclerotic plaque in ApoE^-/-^ mice. The surface of atherosclerotic lesion was decreased significantly (*P* < 0.001) with the overexpression of adiponectin: the percentage of surface lesion in 4 weeks was 27.78 ± 8.64 vs 33.02 ± 5.18 (%); 8 weeks was 31.58 ± 5.87 vs 52.16 ± 5.79 (%), respectively (Fig. 1a, b, c, d, e). The lesion formation in aortic root in adiponectin group mice was inhibited significantly (*P* < 0.01): At 4 week of post-transfection the plaque lesion areas were 1.50 ± 0.74 × 10^4^ μm^2^ in control mice vs. 1.35 ± 0.17 × 10^4^ μm^2^ in adiponectin group mice, and at 8 weeks the plaque lesion areas were 1.70 ± 0.34 × 10^4^ μm^2^ in adiponectin group mice vs. 2.21 ± 0.28 × 10^4^ μm^2^ in control mice; lesion lumen ratios were decreased significantly (*P* < 0.001): lesion lumen ratio in 4 weeks was 28.36 ± 3.22 in adiponectin group mice vs 35.61 ± 7.43 (%) in control mice, and 8 weeks was 38.25 ± 6.30 vs 56.89 ± 6.21 (%), respectively (Fig. 2a, b, c).

**Fig. 3:**
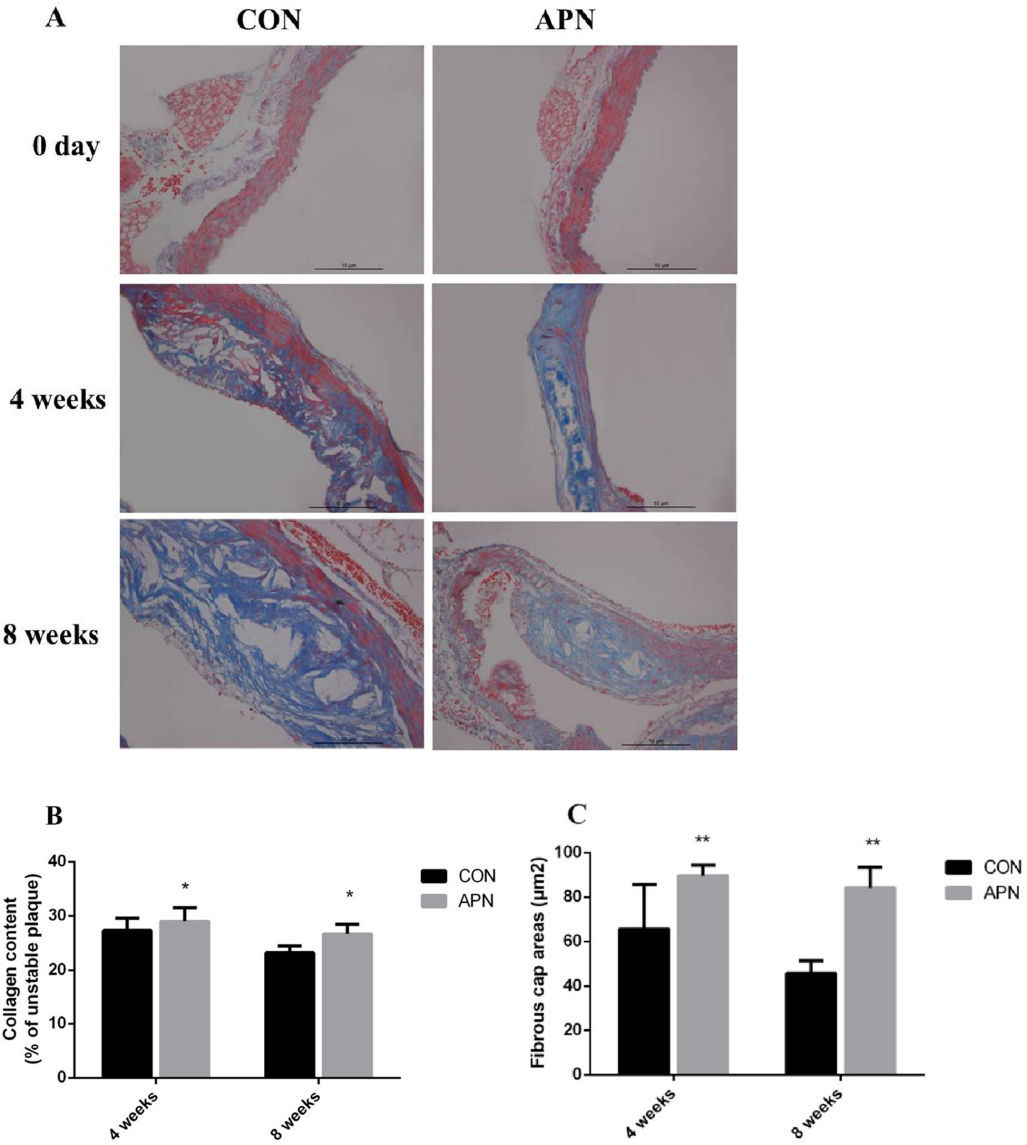
Composition of cells in the aortic arch root **a** Masson trichrome staining of aortic root (×200) **b** Collagen content in advanced atherosclerotic plaques **c** Fibrous cap thickness. Notes: compared with CON group, *P < 0.01; **P < 0.001, *n* = 6, mean ± SD

Advanced plaques lead to plaque rupture [14]. We analyzed collagen contents and fibrous cap thickness of advanced lesions at the level of aortic root. Mice were fed a high-fat diet for 8 weeks, and histological sections were examined after Masson trichrome staining. We found a significant increase in collagen content (*P* < 0.01) (Fig. 3a, b) and fibrous cap thickness of lesions (*P* < 0.001) (Fig. 3a, c) in the adiponectin group mice compared with control group mice.

**Fig. 4:**
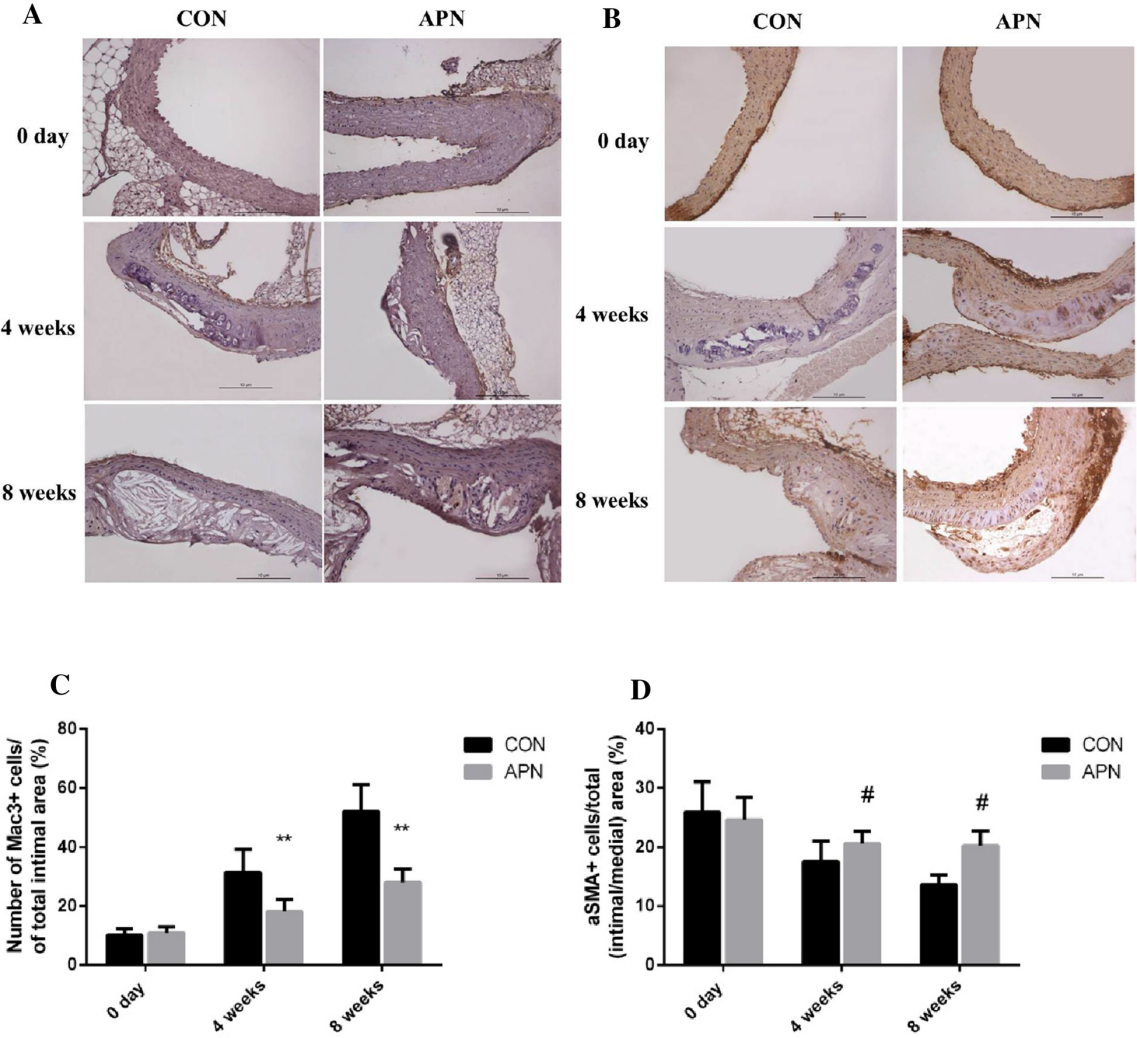
Composition of cells in the aortic arch root **a** The area of atherosclerotic plaques in sections of the proximal aorta was stained with an antibody Mac3 against macrophages (×200) **b** The area of atherosclerotic plaques in sections of the proximal aorta was stained with an antibody against αSMA (×200) **c** the number of Mac3+ cells/% of total intimal area **d** the number of αSMA+ cells/% of total (intimal/medial) area. Notes: compared with CON group, #*P* < 0.05; **P < 0.001, *n* = 6, mean ± SD

Next we examined composition of macrophages (anti-Mac3) and smooth muscle cells(anti-α-smooth muscle actin) in the lesions of ApoE^-/-^ mice aortic root (Fig. 4). Increased macrophage content (*P* < 0.001) in lesions of control group mice compared with adiponectin group mice were detected. In contrast, the relative smooth muscle cell content was significantly less (*P* < 0.05) in the total lesions of control group mice, which represents the composition of vulnerable plaques.

### Overexpression of adiponectin attenuates body weight increase, hyperlipidemia and MMP-9 level

**Fig. 5:**
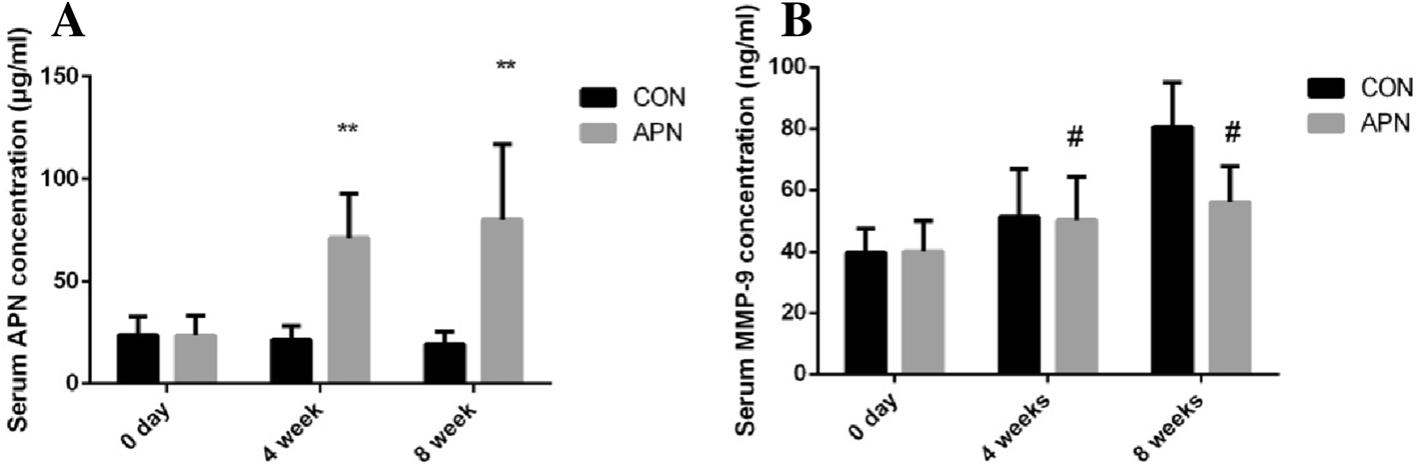
Level of serum adiponectin、MMP-9 **a** Serum concentration of adiponectin. **b** Serum concentration of MMP-9. Notes: compared with CON group, #*P* < 0.05; **P < 0.001, *n* = 12, mean ± SD

The adenovirus mediated adiponectin were delivered by tail vein injection. As shown in Fig. 5a, the expression of adiponectin in the serum was ≈ 4-fold higher than that in the control group. The expression of adiponectin attenuated the increases of serum TC (*P* < 0.001), TG (*P* < 0.001), and LDL-C (*P* < 0.001) induced by the high-fat diet, and attenuated body weight increasing (*P <* 0.05) (Table 2). As increasing serum adiponectin, the level of MMP-9 were significantly decreased (*P* < 0.05): at 4 week, 50.23 ± 14.24 ng/ml in adiponectin group vs. 51.33 ± 15.66 ng/ml in control group, and at 8 week, 55.99 ± 11.74 ng/ml vs. 80.50 ± 14.54 ng/ml (Fig. 5b). This atherosclerosis improvement was found to be due, at least in part, to downregulation of inflammation state by increasing serum adiponectin and decreasing MMP-9.

**Table 2:** Mean body weight and TC, TG, LDL-C, HDL-C in serum

Group	Body weight (g)	TC (mmol/L)	TG (mmol/L)	LDL-C (mmol/L)	HDL-C (mmol/L)
CON 0d	22.79 ± 1.72	7.27 ± 3.51	0.61 ± 0.34	0.79 ± 0.46	0.95 ± 0.41
APN 0d	24.13 ± 3.11	8.01 ± 3.07	0.49 ± 0.24	0.96 ± 0.52	1.38 ± 1.18
CON 4w	27.50 ± 3.31	32.43 ± 10.77	1.18 ± 0.31	10.14 ± 1.48	1.29 ± 0.55
APN 4w	26.04 ± 2.07*	28.66 ± 8.11**	1.03 ± 0.39***	8.06 ± 1.74***	0.91 ± 0.33
CON 8w	35.49 ± 3.41	38.12 ± 6.36	2.01 ± 0.77	10.95 ± 3.23	1.86 ± 1.60
APN 8w	29.95 ± 5.53*	24.54 ± 8.29**	1.00 ± 0.30***	7.57 ± 1.46***	1.07 ± 0.26

Notes: compared with CON group, *P < 0.05; **P < 0.01; ***P < 0.001, *n* = 12, mean ± SD

### Delivery of adiponectin suppressed the activation of NF-κB p65, decreased gene and protein expression of inflammation

**Fig. 6:**
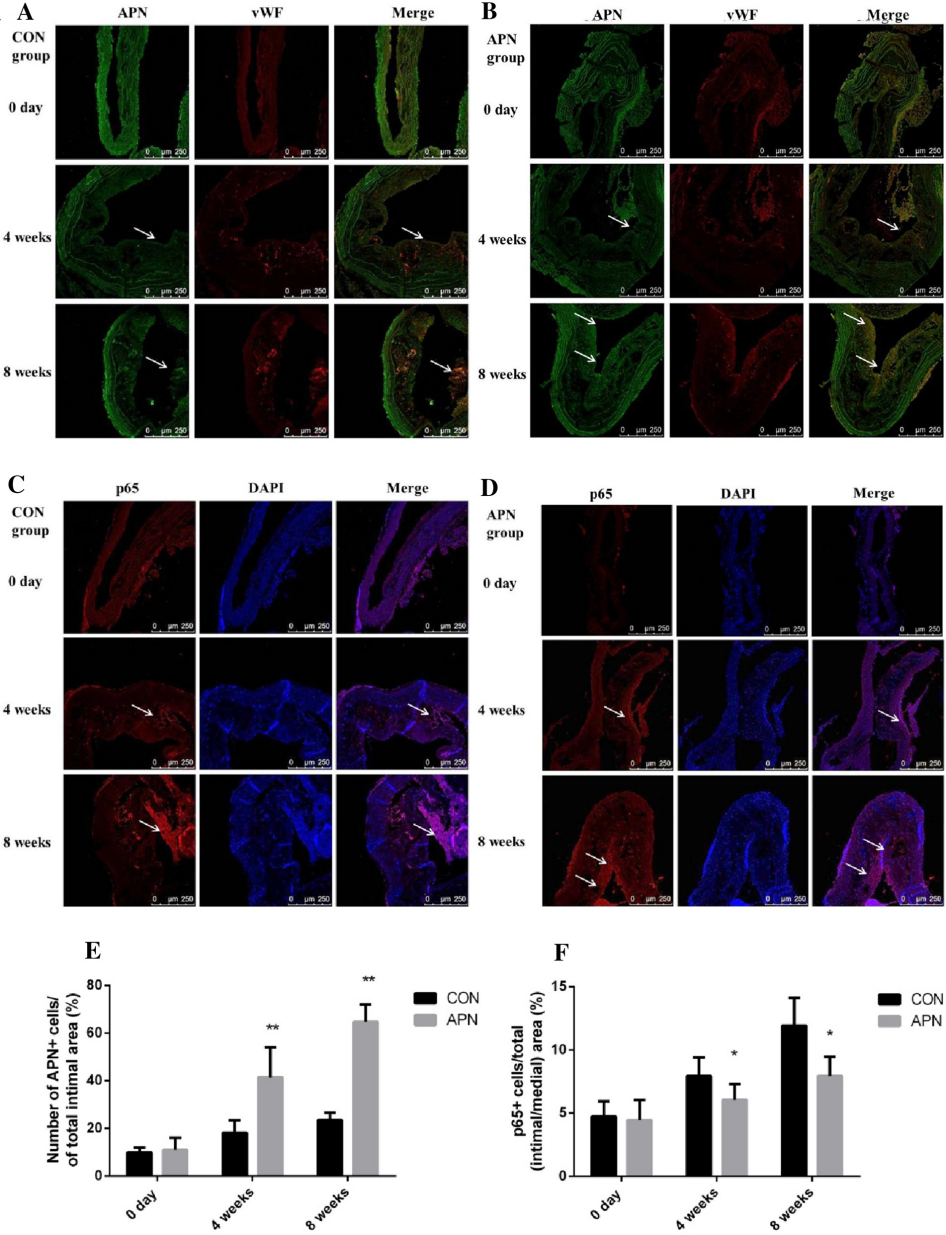
**a** Nuclear NF-κB p65 expression in the aortic arch root in control group **b** Nuclear NF-κB p65 expression in the aortic arch root in adiponectin group **c** Expression of adiponectin、vWF in the vascular aortas in control group (×200) **d** Expression of adiponectin、vWF in the vascular aortas in adiponectin group (×200) **e** the number of APN+ cells/% of total intimal area **f** the number of nuclear NF-κB p65+ cells/% of total (intimal/medial) area. Notes: compared with CON group, *P < 0.01; **P < 0.001, *n* = 6, mean ± SD

**Fig. 7:**
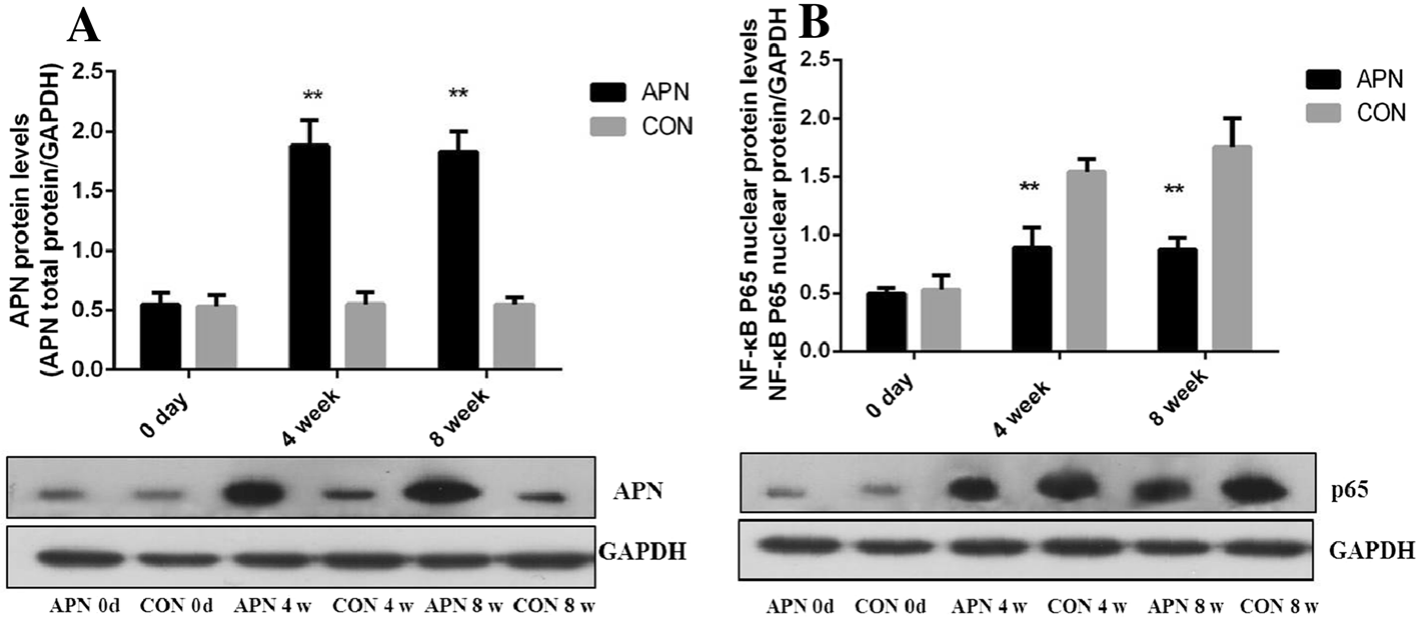
**a** Adiponectin protein expression in the aorta **b** NF-κB p65 protein of nuclear expression in the aorta. Notes: compared with CON group, **P < 0.001, *n* = 3, mean ± SD

Exogenous adiponectin delivery improves inflammation of atherosclerosis by suppressing the activation of NF-κB pathway. Adiponectin effectively inhibits the expression of NF-κB nuclear protein p65. Compared with control group, expression of adiponectin in aortic root endothelial cells was increased in adiponectin group. Immunofluorescence test suggested that the expression of NF-κB p65 increased gradually with the prolongation of the high fat diet time in control group. The expression of the adiponectin increased and the expression of NF-κB p65 decreased in the adiponectin group (*P* < 0.001) (Figs. 6 and 7).

**Fig. 8:**
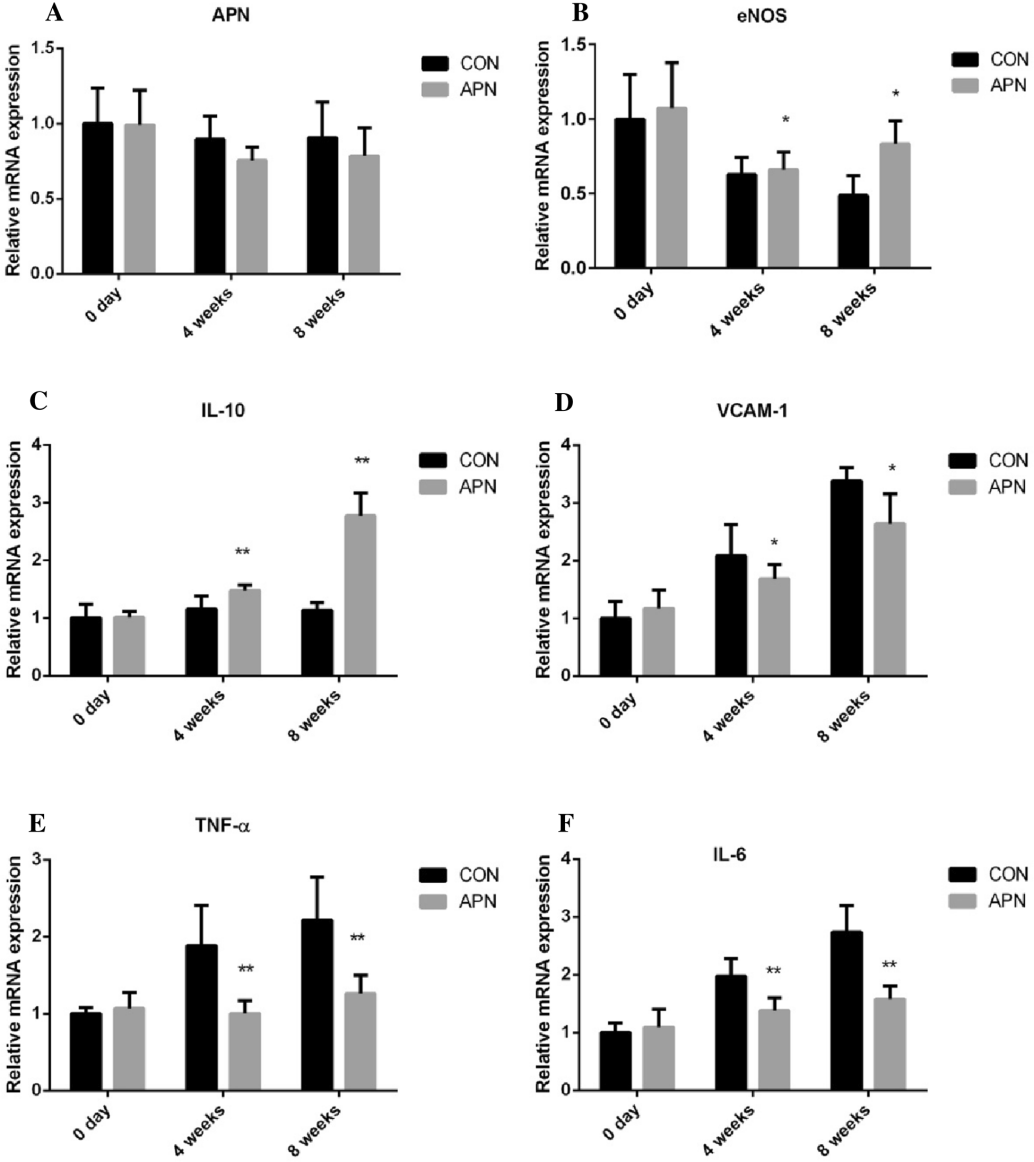
Exogenous adiponectin reduces atherosclerosis-induced inflammation in the aorta **a** mRNA expression of adiponectin in the vascular aortas of mice **b** mRNA expression of eNOS **c** mRNA expression of IL-10 **d** mRNA expression of VCAM-1 **e** mRNA expression of TNF-α **f** mRNA expression of IL-6. Notes: compared with CON group, *P < 0.01; **P < 0.001, *n* = 6, mean ± SD

**Fig. 9:**
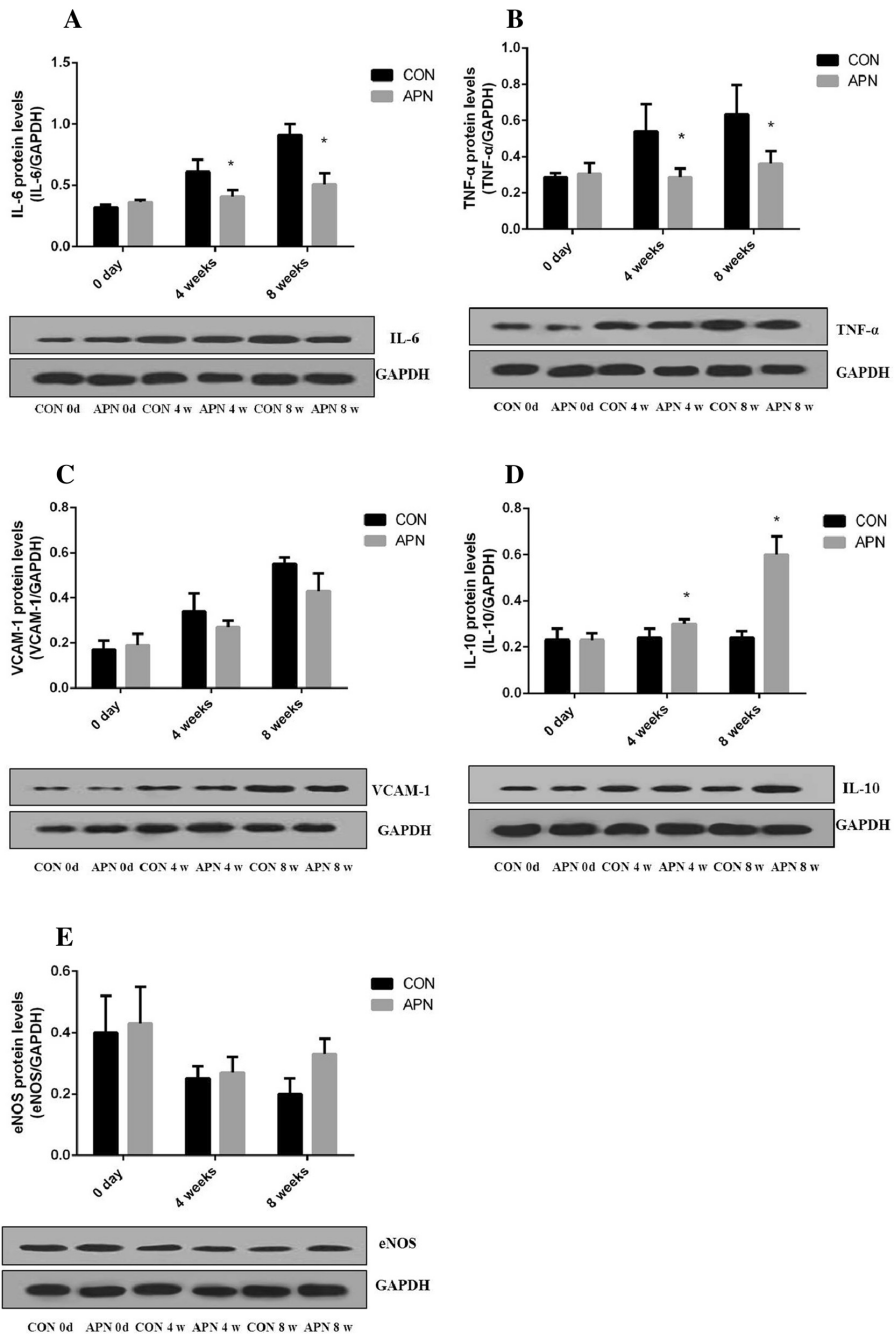
Exogenous adiponectin reduces atherosclerosis-induced inflammation in the aorta **a** protein expression of IL-6 in the vascular aortas **b** protein expression of TNF-α **c** protein expression of VCAM-1 **d** protein expression of IL-10 **e** protein expression of eNOS. Notes: compared with CON group, *P < 0.01, *n* = 3, mean ± SD

The exogenous adiponectin increased the mRNA expression of the anti-inflammatory factors eNOS (*P* < 0.05) and IL-10 (*P* < 0.001), and reduced the mRNA expression of inflammatory factors TNF-α (*P* < 0.001), IL-6 (*P* < 0.001), VCAM-1 (*P* < 0.05), respectively. Adiponectin effectively reduced proinflammatory factors gene expression, indicating that adiponectin can attenuate the inflammatory reactions relevant to atherosclerosis (Figs. 8 and 9).

## Discussion

Adiponectin deficiency leads to increased oxidative stress and inflammation under high fat diet conditions in type 2 diabetic mice [13]. NF-κB has also been regarded as a proatherogenic factor, mainly because of its regulation of a variety of the proinflammatory genes linked to atherosclerosis [15]. Importantly, in current study, exogenous adiponectin delivery improves inflammation of atherosclerosis effectively by inhibiting the expression of NF-κB nuclear protein p65 and proinflammatory factors regulated by NF-κB. The major findings of the current work were as follows: 1) Exogenous supplementation of adiponectin using adenovirus was found to improve atherosclerosis. 2) This improvement was found to be due, at least in part, to downregulation of inflammation state by increasing serum adiponectin and decreasing MMP-9. 3) Exogenous supplementation of serum protein adiponectin was found to normalize dyslipidemia by reducing serum TC, TG, and LDL-C. Mice body weight decreased significantly as the adiponectin level increased. 4) Exogenous adiponectin delivery improves inflammation of atherosclerosis effectively by inhibiting the expression of NF-κB nuclear protein p65 and inflammatory factors regulated by NF-κB.

### Adiponectin inhibited the formation of atherosclerosis

Atherosclerosis is regarded as an inflammatory disease. Experimental studies have indicated that adiponectin has potential anti-atherogenic and anti-inflammatory properties [16]. In the current study, the formation of atherosclerosis plagues was inhibited by adiponectin. The adiponectin group showed far smaller damaged areas than those of control group in 4 and 8 weeks. It is suggested that adiponectin prevents and slows the progression of atherosclerosis. When the vascular endothelium is injured, adiponectin accumulates in the subintimal space of the arterial wall through its interaction with collagens in the vascular intima [17]. Adiponectin inhibited TNF-α-induced monocyte adhesion and expression of endothelial-leukocyte adhesion molecule-1, E-selectin, VCAM-1, and ICAM-1 on the endothelium [18]. Previous studies have shown that elevated plasma adiponectin suppresses the development of atherosclerosis in vivo [19]. Fourteen days after Ad-APN treatment, the lesion formation in aortic sinus was inhibited by 30 % in Ad-APN treated ApoE^-/-^ mice compared with control mice. The lipid droplets became smaller than in control mice [20]. Our previous studies showed that as the dose of adiponectin increased, atherosclerosis-induced damage gradually eased [21]. In a recent paper of the Scherer’s group, genetically manipulated adiponectin levels in traditional rodent models (adiponectin knockout mice or mice crossed with the low-density lipoprotein receptor-null and the apoliprotein E-null mouse models) did not correlate with a suppression of the atherogenic process [11]. The lack of a phenotype in gain- and loss of function study in mice suggests a lack of causation for adiponectin in inhibiting the buildup of atherosclerotic lesions [22]. These data indicate that the actions of adiponectin on the cardiovascular system are complex and multifaceted. These discrepancies may have been caused by differences in strain, age, and disease status among the animals and among the dose and effective concentration of adiponectin [23].

### Overexpression of adiponectin attenuates body weight increase, hyperlipidemia and MMP-9 level

Lipid accumulation causes dysregulation of adipokine production, which makes a strong contribution to the onset of inflammation and atherosclerosis [24]. The atherogenic process is initiated by the formation of oxidized LDL-cholesterol in the arterial wall, which causes recruitment of circulating monocytes and differentiation into macrophages [25]. Clinical studies indicate a close association of low adiponectin levels with many obesity-related disorders [26]. Adiponectin which has anti-hyperglycemic, anti-atherogenic, and anti-inflammatory properties, could have important clinical benefits in terms of development of therapies for the prevention and for the treatment of obesity and obesity-related diseases [27]. In the current study, exogenous adiponectin supplementation significantly reduced TC,TG, LDL-C without reducing HDL-C levels. One possible reason for this is that ApoE, a major apolipoprotein, acts as a ligand for low-density lipoprotein and very low density lipoprotein receptors in the liver. Atherogenic metabolic disturbances may not always be adequately reflected by the level of HDL-C. This was because VLDL contained more cholesterol and the relative cholesterol content of VLDL increased across TG quintiles [28]. In the current study, exogenous adiponectin supplementation significantly reduced body weight gain with HFD feeding. Several lines of evidence from the metabolomic analyses support the concept of adiponectin enhancing mitochondrial function. Adiponectin supplementation induced the coordinated decrease of acetyl-CoA and acylcarnitines and these occurred with normalization of pathological structures in the mitochondria [29]. Pair-feeding revealed that overexpression of globular adiponectin markedly reduced body weight gain of ob/ob mice. These data suggest that overexpression of globular adiponectin increased energy expenditure and consequently increased food intake [30]. Adiponectin serves as a regulator of many important hepatic genes in glucose and lipid biosynthesis and catabolism; the cumulative effects of all these genes expressed at the optimal levels create the basal tone essential for maintaining sensitive insulin responsiveness. For example, increased free fatty acids (FFAs) in plasma induce insulin resistance. Adiponectin promotes the expression of key hepatic genes in triglyceride formation and lipid clearance, which promotes FFA clearance from plasma and to decreased plasma FFAs [31]. MMP-9 is a perfect biomarker of atherosclerosis. MMP-9 is overexpressed in progressive atherosclerotic plaques obtained from humans undergoing endarterectomy and is especially prevalent in the cap regions where macrophages accumulate, implicating macrophages and MMP-9 in plaque rupture [32]. The previous study demonstrated an increased risk of severe atherosclerosis and unstable plaques in patients with higher MMP-9 levels [33]. Matrix (ECM) components of the atherosclerotic plaque, including collagen, elastin, and proteoglycans, and as such is a critical modulator of plaque stability [34]. Increased MMP-9 was shown in advanced atherosclerotic lesions in the Ldl4^-/-^Apob100/100 mouse model [35]. In the current study, there was significantly less expression of MMP-9 in adiponectin treated group in 8 weeks with the increase of the adiponectin (*P* < 0.01*)*. This atherosclerosis improvement was found to be due, at least in part, to downregulation of inflammation state by increasing serum adiponectin and decreasing MMP-9.

### Delivery of adiponectin suppressed the activation of NF-κB p65 and proinflammatory gene expression

In current study, exogenous adiponectin increased anti-inflammatory factor eNOS and IL-10 mRNA expression, reduced the expression of pro-inflammatory factor TNF-α, IL-6, VCAM-1 mRNA expression. Adiponectin effectively inhibited the activation of NF-κB pathway by inhibiting the expression of NF-κB nuclear protein p65. NF-κB is a key transcriptional regulator of most inflammatory genes and is widely believed to trigger both the onset and resolution of inflammation by coordinating the expression of a wide variety of genes that control inflammatory and immune responses. Constitutive activation of NF-κB is often associated with inflammatory diseases such as rheumatoid arthritis, inflammatory bowel disease, multiple sclerosis and asthma [36]. In others vitro study, adiponectin suppressed TNF-α–induced IκBα phosphorylation and subsequent NF-κB activation without affecting other TNF-α mediated phosphorylation signals, including Jun N-terminal kinase, p38 kinase, and Akt kinase. These observations raise the possibility that adiponectin, which is naturally present in the blood stream, modulates the inflammatory response of endothelial cells through cross talk between cAMP-PKA and NF-κB signaling pathways [37]. Prior work had shown that adiponectin inhibited endothelial cell dysfunction through stimulation of endothelial nitric oxide and endothelium-dependent vasodilation, inhibition of cytokine, chemokine, and adhesion molecule expression [38]. These findings might indicate that, in inflammation, NF-κB stimulating pathways of adiponectin are likely suppressed and anti-inflammatory signals are transduced.

## Conclusions

To summarize, in current study, it was demonstrated that adiponectin effectively inhibits the activation of NF-κB pathway by inhibiting the expression of NF-κB nuclear protein p65. To elucidate the adiponectin to reduce this inflammation in atherosclerosis effectively may be involved in inhibiting the NF-κB pathway. In this way, in addition to the treatment of basic diseases, restoring levels of adiponectin and the integrity of the vascular tissue by reducing the inflammation. It may promote pathological and physiological studies of weight-related diseases and in-depth studies of the mechanism by which adiponectin regulates inflammation. Collectively, these findings give new insights into the linkage between adiponectin and inflammation in the course of atherosclerosis, but the most effective dose of adiponectin has not been identified. The mechanism by which adiponectin regulates inflammation is still not very clear. Further studies are needed to evaluate the therapeutic potential of these observations.
